# Efficacy and safety of Eribulin-based chemotherapy in HER2 negative advanced breast cancer patients: a real-world study

**DOI:** 10.3389/fonc.2025.1499701

**Published:** 2025-06-06

**Authors:** Yuting Li, Dan Han, Ting Hu, Jie Xiong, Yuehua Wang, Yanxia Zhao

**Affiliations:** ^1^ Cancer Center, Union Hospital, Tongji Medical College, Huazhong University of Science and Technology, Wuhan, China; ^2^ Institute of Radiation Oncology, Union Hospital, Tongji Medical College, Huazhong University of Science and Technology, Wuhan, China; ^3^ Hubei Key Laboratory of Precision Radiation Oncology, Wuhan, China; ^4^ Department of Oncology, Xiangyang Central Hospital, Xiangyang, China

**Keywords:** Eribulin, breast cancer, HER2-low, combination therapy, progression-free survival

## Abstract

**Background:**

Breast cancer is recognized as one of the most common cancers worldwide, exhibiting a notably high incidence rate among women in China. Despite significant advancements in therapeutic approaches, the prognosis for patients diagnosed with advanced stages of the disease remains poor. Therefore, there is an urgent necessity to investigate the effectiveness and safety of treatments such as Eribulin, a non-taxane microtubule inhibitor that is recommended for use beyond second-line therapy.

**Methods:**

This retrospective multicenter study assessed 105 patients with HER2-negative advanced breast cancer who received Eribulin treatment from 2020 to 2023.

**Results:**

With a median follow-up of 13.3 months, the median progression-free survival (PFS) was 6.0 months. Patients on early-line Eribulin had a significantly longer PFS (6.7 vs. 4.9 months, P = 0.038) than those on later lines. Combination therapy tended toward longer PFS than monotherapy (6.4 vs. 4.9 months; P = 0.319), albeit non-significantly. Combinations with PD-1 inhibitors or chemotherapy had a higher PFS than those with anti-angiogenic agents (P = 0.022). Among ER-negative patients in the combination therapy subgroup, HER2-zero tumors had a significantly longer PFS than HER2-low tumors (9.5 vs. 4.1 months, P = 0.026). The most frequently observed adverse events were hematological toxicity, with the majority classified as manageable in severity.

**Conclusion:**

The findings emphasize the potential of Eribulin in the treatment of HER2-negative advanced breast cancer, highlighting the need for further large-scale, prospective studies to refine and enhance treatment strategies.

## Introduction

Breast cancer, recognized as a prevalent malignant neoplasm with considerable global ramifications, ranked as the second most frequently diagnosed cancer in 2022 (1). In the context of China, it occupied the fifth position among all cancer types, yet it emerged as the foremost cause of cancer-related mortality among women during the same year (2). Despite advancements in treatment strategies, advanced breast cancer continues to present a challenge, with many patients facing short survival periods (3, 4). For inoperable advanced cases, drug treatment remains the predominant therapeutic approach (5).

Eribulin, a non-taxane microtubule inhibitor, has shown great promise in extensive clinical studies. It has been demonstrated to significantly extend both progression-free survival (PFS) and overall survival (OS), especially when administered as a treatment beyond the second line for advanced breast cancer (6, 7). However, despite its proven efficacy, the real-world application and utilization patterns of Eribulin among breast cancer patients in China have not been well-documented. This gap in knowledge highlights an important area that urgently requires further research to optimize its clinical use and patient outcomes. Specifically, identifying more suitable patient populations and exploring optimal combination treatment regimens in real-world settings, as well as providing a basis for further large-scale clinical trials, are pressing issues that demand immediate attention. These endeavors are crucial for maximizing the therapeutic potential of Eribulin and improving the prognosis of breast cancer patients.

Human epidermal growth factor receptor 2 (HER2) overexpression or amplification has long defined a high-risk molecular subtype of breast cancer, characterized by aggressive tumor progression and poor patient prognosis (8). This biomarker has served as a cornerstone for identifying patients who can benefit from classical anti-HER2 therapies, such as trastuzumab, transforming what was once a dismal diagnosis into a more manageable condition (9). HER2-negative breast cancer exhibits significant biological heterogeneity, particularly between HER2-low (IHC 1+ or 2+/*in situ* hybridization assay, ISH not amplified) and HER2-zero (IHC 0) subtypes (10). However, recent clinical research advancements have challenged the traditional binary HER2 classification, revealing a more intricate understanding of HER2-related breast cancer subtypes. A significant breakthrough lies in the identification of HER2-low breast cancer as a distinct, therapeutically targetable entity, defined by low-level HER2 expression and validated by trials of novel anti-HER2 antibody-drug conjugates. This discovery emphasizes the biological heterogeneity within HER2-negative breast cancer, as HER2-low tumors show sensitivity to next-generation anti-HER2 antibody-drug conjugates such as trastuzumab deruxtecan (11). This dichotomy not only reshapes our understanding of HER2 biology but also calls for a shift in treatment strategies.

Numerous studies have consistently shown that HER2-low breast cancer presents distinct clinical characteristics when compared to HER2-zero breast cancer. Specifically, it demonstrates a significantly higher incidence of hormone receptor (HR) positivity, lower nuclear and histologic grades, and a reduced Ki-67 proliferation index, indicating relatively less aggressive biological behavior (12).

Although preclinical studies have proposed that bidirectional crosstalk between the HER2 and HR pathways may drive endocrine resistance (13), the clinical impact of HER2-low status on the efficacy of CDK4/6 inhibitors has yielded conflicting results. A pooled analysis of nine studies revealed that patients with HER2-low tumors had a significantly elevated risk of progression and/or death compared to those with HER2-zero tumors (14). Prospective clinical trials have shown that, among HR-positive patients, the combination of CDK4/6 inhibitors and endocrine therapy significantly prolongs PFS in HER2-low patients, but not in HER2-zero patients (15). This discrepancy highlights the complexity of translating preclinical findings to clinical practice, underscoring the need for further research to elucidate the underlying mechanisms and optimize treatment strategies for patients with different HER2 expression profiles.

Several studies have explored the response of HER2-low breast cancer to cytotoxic drugs. In neoadjuvant settings for operable breast cancer patients, HER2-low status has shown relatively consistent predictive value for pathological complete response (pCR) and disease-free survival (DFS). Multiple meta-analyses have demonstrated that, regardless of hormone receptor status, patients with HER2-low tumors have a significantly lower pCR rate compared to those with HER2-zero tumors (16, 17). Moreover, in terms of survival outcomes, meta-analysis indicated that HER2-low breast cancer patients tend to have longer OS and DFS than their HER2-zero counterparts (16, 17). However, the relationship between HER2 expression and treatment efficacy is uncertain in advanced breast cancer patients. For instance, in triple-negative breast cancer patients receiving first-line platinum-based chemotherapy, no significant differences in PFS and OS were observed between HER2-low and HER2-zero patients (18). Similarly, another study on advanced breast cancer patients undergoing chemotherapy found no clinically meaningful differences in PFS or OS based on HER2-low versus HER2-zero status (19). Therefore, some studies suggest that HER2-low breast cancer does not exhibit distinct biological behaviors in response to chemotherapy (19, 20).

As the role of HER2-low status in predicting the response to chemotherapy remains controversial, with inconsistent findings between operable and advanced breast cancer patients, the treatment landscape for HER2-negative advanced breast cancer, especially in the Chinese population, needs further clarification. Despite the potential of drugs like Eribulin, a non-taxane microtubule inhibitor, in improving survival outcomes for advanced breast cancer patients, there is a dearth of large-scale, real-world evidence specific to Chinese patients with HER2-negative advanced breast cancer.

In this study, we aim to bridge the knowledge gap by conducting real-world research on the efficacy and safety of Eribulin in treating HER2-negative advanced breast cancer in Chinese patients. Given the limited large-scale clinical data specific to this population, our research seeks to provide evidence-based insights to optimize treatment strategies.

## Methods

### Study population

This retrospective real-world study was conducted across multiple centers. A total of 174 patients with advanced breast cancer treated at two hospitals between January 2020 and December 2023 were screened. The criteria for inclusion encompassed individuals aged 18 years or older, female patients, those with HER2-negative status, and participants who underwent a minimum of two cycles of Eribulin therapy during the advanced disease phase within the specified timeframe. Conversely, patients were excluded if they had concurrent malignancies, incomplete follow-up information, or if they were lost to follow-up. The study received approval from the Ethics Committee of Union Hospital, Tongji Medical College (Approval number: UHCT21562), which also provided a waiver for the necessity of obtaining informed consent from the participants.

### Treatment

All patients received at least two cycles of Eribulin treatment, and some were also treated with Eribulin in combination with other therapies such as anti-angiogenic drugs, PD-1 inhibitors, or alternative chemotherapy regimens. Eribulin was administered on days 1 and 8, with each cycle consisting of 21 days for most patients. Some patients received Eribulin on days 1 and 15, with a cycle duration of 28 days. The efficacy and tolerability of the biweekly eribulin regimen have been validated by Kobayashi et al (21). and Ohtani et al (22).

### Data collection

Data collection included patient characteristics (age, sex), tumor characteristics (date of diagnosis, number, and sites of metastases including central nervous system metastases), medical history (prior systemic treatments, previous chemotherapy regimen, and exposure to endocrine therapy), details of eribulin treatment (start and end dates, combination regimens), treatment efficacy (evaluated using RECIST v1.1 criteria), and survival outcomes. The data cut-off was March 8, 2024.

### Outcomes

The primary endpoint was PFS in the entire study population, defined as the time from treatment initiation to disease progression. Secondary endpoints included objective response rate (ORR) and disease control rate (DCR) based on RECIST criteria v1.1, investigation of prognostic and predictive factors, and safety assessment. Treatment efficacy was evaluated every two to three cycles using computed tomography or magnetic resonance imaging at baseline and during treatment. Efficacy was assessed based on Response Evaluation Criteria in Solid Tumors version 1.1. Treatment-related adverse events (AEs) were assessed through clinical evaluation, complete blood cell count, and full serum chemistry, graded according to Common Terminology Criteria for Adverse Events version 5.0.

### Statistical analysis

Descriptive statistics summarized patient data, including frequency and percentages for categorical variables, and median and range for continuous variables. Statistical analysis was conducted using SPSS 23.0 software. Continuous variables were presented as a median and interquartile range, while categorical variables were presented as numbers and percentages. For categorical variables, either the Chi-square test or Fisher’s exact test was utilized. Survival curves for PFS were generated using the Kaplan-Meier method, with the calculation of median PFS and its 95% confidence interval (95% CI). The log-rank test served as the method for comparing Kaplan-Meier survival distributions. Cox regression analysis was used to examine potential factors associated with PFS. A two-sided p-value <0.05 indicated statistical significance.

## Results

### Baseline characteristics

We included 105 patients in the final analysis based on predefined inclusion and exclusion criteria (**Table 1**). The primary reasons for exclusion were: Incomplete follow-up data (n = 34), HER2-positive (n = 23), switching to other regimens for maintenance treatment after induction treatment with eribulin, such as endocrine therapy, PD-1 inhibitor, or capecitabine (n = 12). The median age was 53.2 years (range 30.0–86.0 years). Among them, 54 patients (51.4%) received eribulin as first- or second-line therapy. Monotherapy was administered to 45 patients (42.9%), while 60 patients (57.1%) received combination therapy. Sixty-three patients (60.0%) were HER2-low, and 63 patients (60.0%) were hormone receptor (HR) positive. The most common metastatic sites were bone, liver, and lung, with bone metastases present in over half of the patients (56.2%). Prior to receiving eribulin, patients had been treated with Taxanes (55.24%), capecitabine (32.38%), vinorelbine (21.90%), gemcitabine (19.05%), or other drugs. Eribulin was administered to 87 patients using the three-week regimen and to 18 patients using the biweekly regimen.

**Table 1:** Patient characteristics at baseline.

Characteristics	Number of patients (%) (N = 105)
Median age (years, range)	53.2 (30.0-86.0)
ECOG PERFORMANCE-STATUS SCORE	
0	77 (73.33%)
1	22 (20.95%)
2	6 (5.71%)
HORMONE RECEPTOR STATUS	
ER positive	63 (60.00%)
ER negative	42 (40.00%)
HER2 status	
HER2 zero	42 (40.00%)
HER2 low	63 (60.00%)
Ki67 status (%)	
>20	21 (20.00%)
≤20	84 (80.00%)
METASTATIC SITES	
Liver	48 (45.7%)
Lung	49 (46.7%)
Bone	59 (56.2%)
Brain	15 (14.3%)
Pleura	17 (16.2%)
NUMBER OF METASTATIC SITES	
1	25 (23.81%)
2	31 (29.52%)
≥3	49 (46.67%)
CHEMOTHERAPY LINES CONTAINING ERIBULIN	
1st	32 (30.5%)
2nd	22 (21.0%)
3rd or later	51 (48.5%)
PREVIOUS CHEMOTHERAPY DRUG	
Taxanes	58 (55.24%)
Capecitabine	34 (32.38%)
Vinorelbine	23 (21.90%)
Gemcitabine	20 (19.05%)
REGIMEN	
Monotherapy	45 (42.86%)
Combine with anti-angiogenesis therapy	22 (20.95%)
Combine with PD-1 inhibitor or chemotherapy	38 (36.19%)

### Efficacy

**Figure 1:**
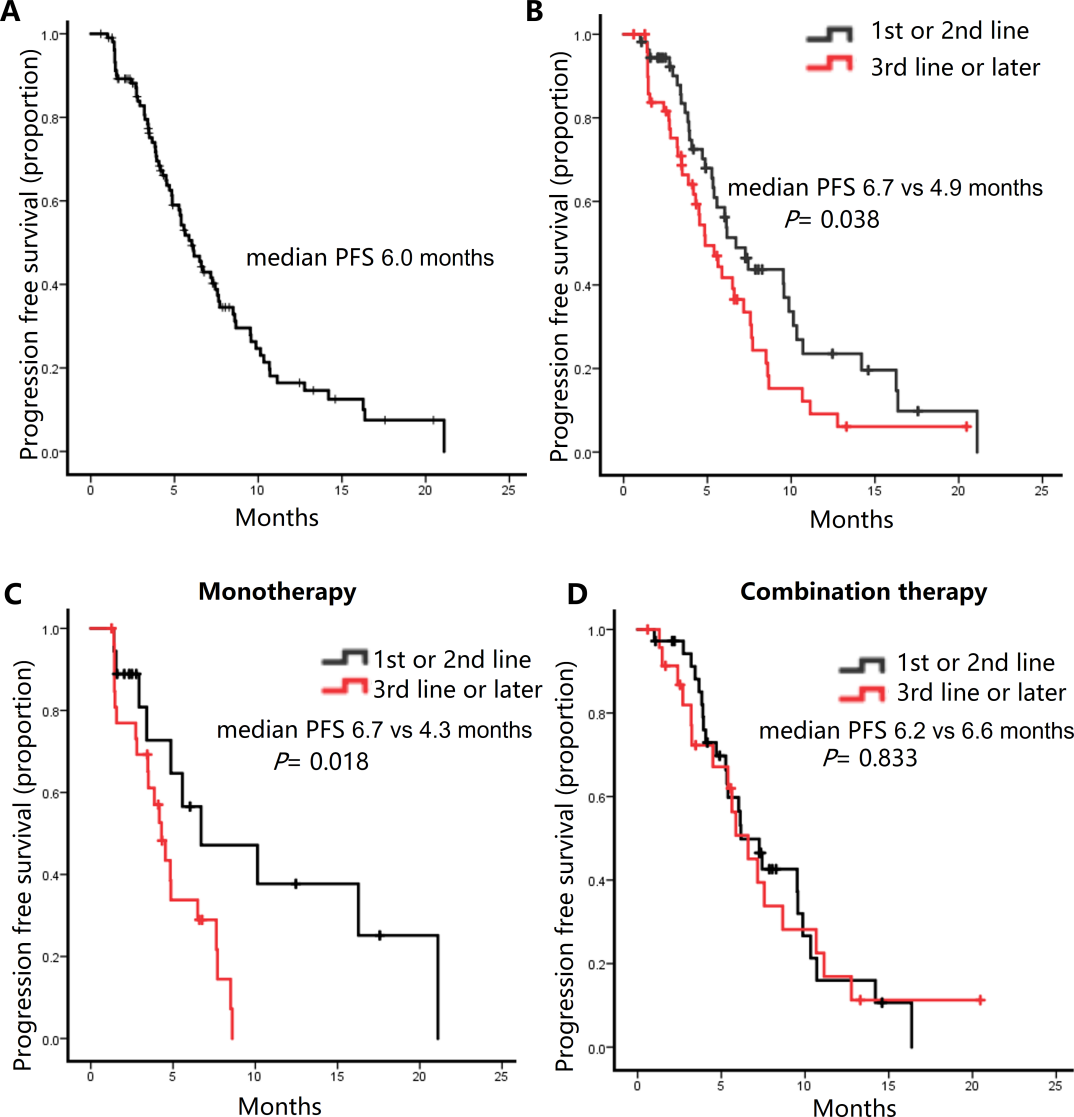
Kaplan-Meier curves for progression-free survival (PFS) in the patients receiving Eribulin-based chemotherapy. **(A)** All patients. **(B)** Comparison of PFS between patients receiving Eribulin as first- or second-line therapy versus Later lines. **(C)** Comparison of PFS between patients receiving combination as first- or second-line therapy versus Later lines. **(D)** Comparison of PFS between patients receiving Eribulin monotherapy as first- or second-line therapy versus Later lines.

Follow-up continued until March 8, 2024, with a median follow-up duration of 13.3 months. The median PFS was 6.0 months, with a range of 3.2 to 11.3 months for all patients (**Figure 1A**). During the study, 31 patients (29.5%) achieved a partial response, 63 patients (60.0%) remained stable, and 11 patients (10.5%) experienced disease progression (**Table 2**).

**Table 2:** Treatment response rate in all patients.

Best overall response	ER- (n=63)	ER+ (n= 42)	HER2-zero (n=42)	HER2-low (n= 63)	1^st^ or 2^nd^ (n=54)	3^rd^ or later (n= 51)	Monotherapy (n= 45)	Anti-angiogenesis (n=22)	PD-1 inhibitor (n= 11)	Chemotherapy (n= 27)
Disease progression	7 (11.1)	4 (9.5)	2 (4.7)	9 (14.3)	3 (5.6)	8 (15.7)	7 (15.6)	3 (13.7)	0 (0.00)	1 (3.7)
Stable disease	38 (60.3)	25 (59.5)	27 (64.3)	36 (57.1)	32 (59.3)	31 (60.8)	28 (62.2)	14 (63.6)	6 (54.5)	15 (55.6)
Partial response	18 (28.6)	13 (31.0)	13 (31.0)	18 (28.6)	19 (35.2)	12 (23.5)	10 (22.2)	5 (22.7)	5 (45.5)	11 (40.7)
ORR	18 (28.6)	13 (31.0)	13 (31.0)	18 (28.6)	19 (35.2)	12 (23.5)	10 (22.2)	5 (22.7)	5 (45.5)	11 (40.7)

#### Efficacy according to prior chemotherapy lines

The clinical response was evaluated based on prior lines of chemotherapy. Among 54 patients receiving eribulin as first- or second-line chemotherapy, 19 patients (35.2%) achieved a partial response (PR), and 32 patients (59.3%) exhibited stable disease (SD). In contrast, among 51 patients receiving eribulin as third-line or later, 12 (23.5%) achieved PR, and 31 (60.8%) had SD (**Table 2**). When eribulin is received in the early treatment line, both the ORR and the DCR tend to be superior to those when eribulin is received in the later treatment line, but there is no statistically significant difference (ORR: P = 0.191; DCR: P = 0.09) (**Supplementary Table 1**). The median PFS was significantly longer in patients receiving eribulin early than later (6.7 vs 4.9 months, P = 0.038) (**Figure 1B**). In the monotherapy subgroup, eribulin administered in the early treatment line demonstrated a significant survival advantage over its use in the late treatment line, with median PFS of 6.7 months and 4.3 months, respectively (P = 0.018) (**Figure 1C**). In contrast, in the combination therapy subgroup, the outcomes between the early and late treatment lines were comparable, with median PFS of 6.2 months and 6.6 months, respectively (P = 0.833) (**Figure 1D**).

#### Efficacy according to combination regimens

**Figure 2:**
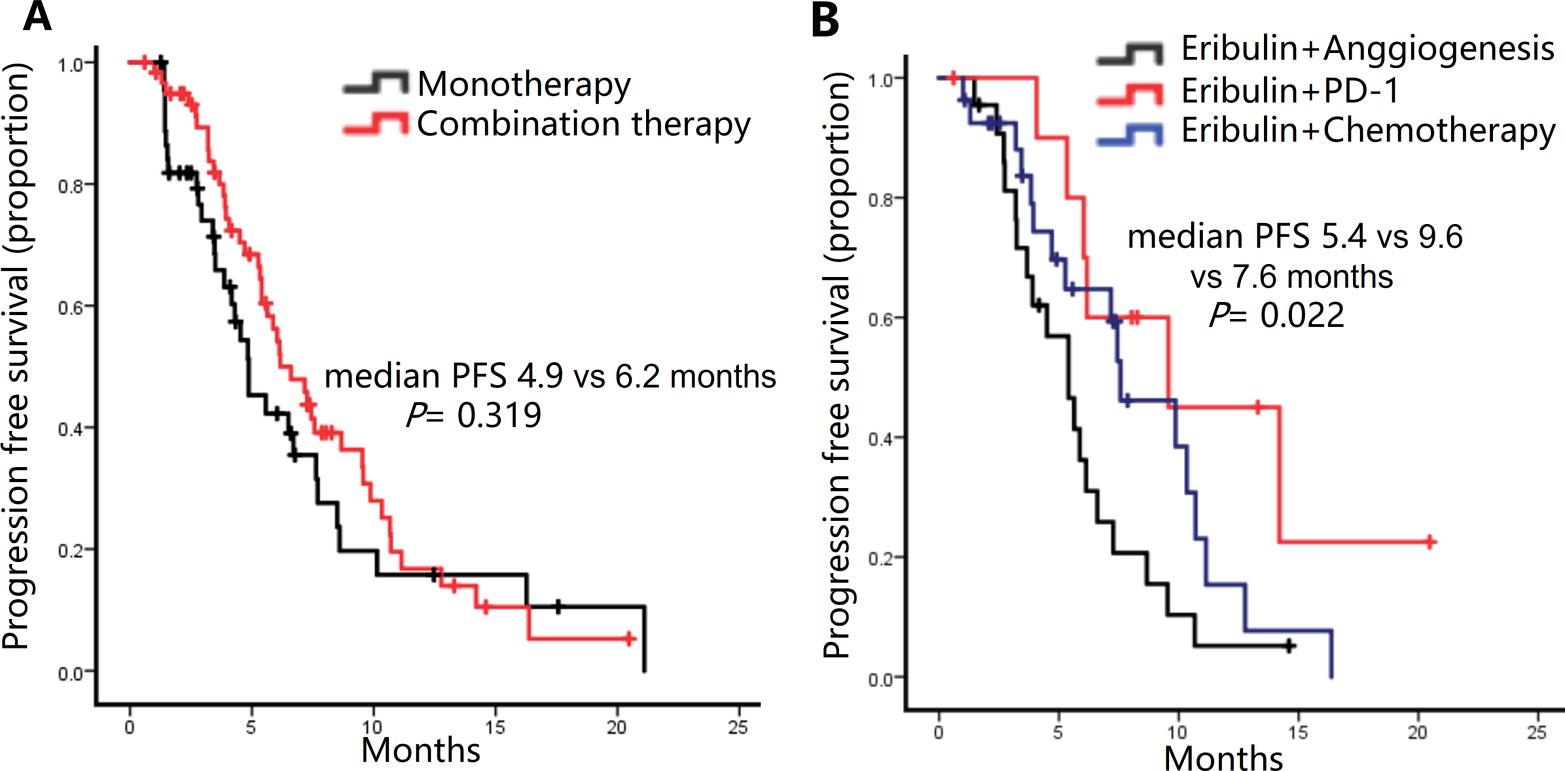
Kaplan-Meier curve of progression-free survival (PFS) of patients treated with different regimens. **(A)** PFS in patients receiving Eribulin alone or combined with other drugs. **(B)** PFS in patients receiving Eribulin combined with anti-angiogenesis drugs, PD-1 inhibitor, or other chemotherapy drugs.

Although not reaching statistical significance, combination therapy appeared to confer a longer median PFS compared to monotherapy, with values of 6.4 months and 4.9 months, respectively (P = 0.319) (**Figure 2A**). Anti-angiogenic agents (anlotinib or bevacizumab) were predominant in combination therapies (21.0% of patients). PD-1 monoclonal antibodies (11 cases) or chemotherapy (27 cases, e.g., carboplatin or capecitabine) trended toward higher PFS than anti-angiogenic agents (9.6 vs. 7.6 vs. 5.4 months; P = 0.022) (**Figure 2B**). The ORR of the combination with a PD-1 inhibitor (45.5%) or chemotherapy (40.7%) was superior to that of the monotherapy group (22.2%) and the combination with anti-angiogenesis agents (22.7%). There is also a similar trend in the DCR. However, there were no statistically significant difference (ORR: P = 0.202; DCR: P = 0.253) (**Supplementary Table 2**).

#### Efficacy according to HER2 status

**Figure 3:**
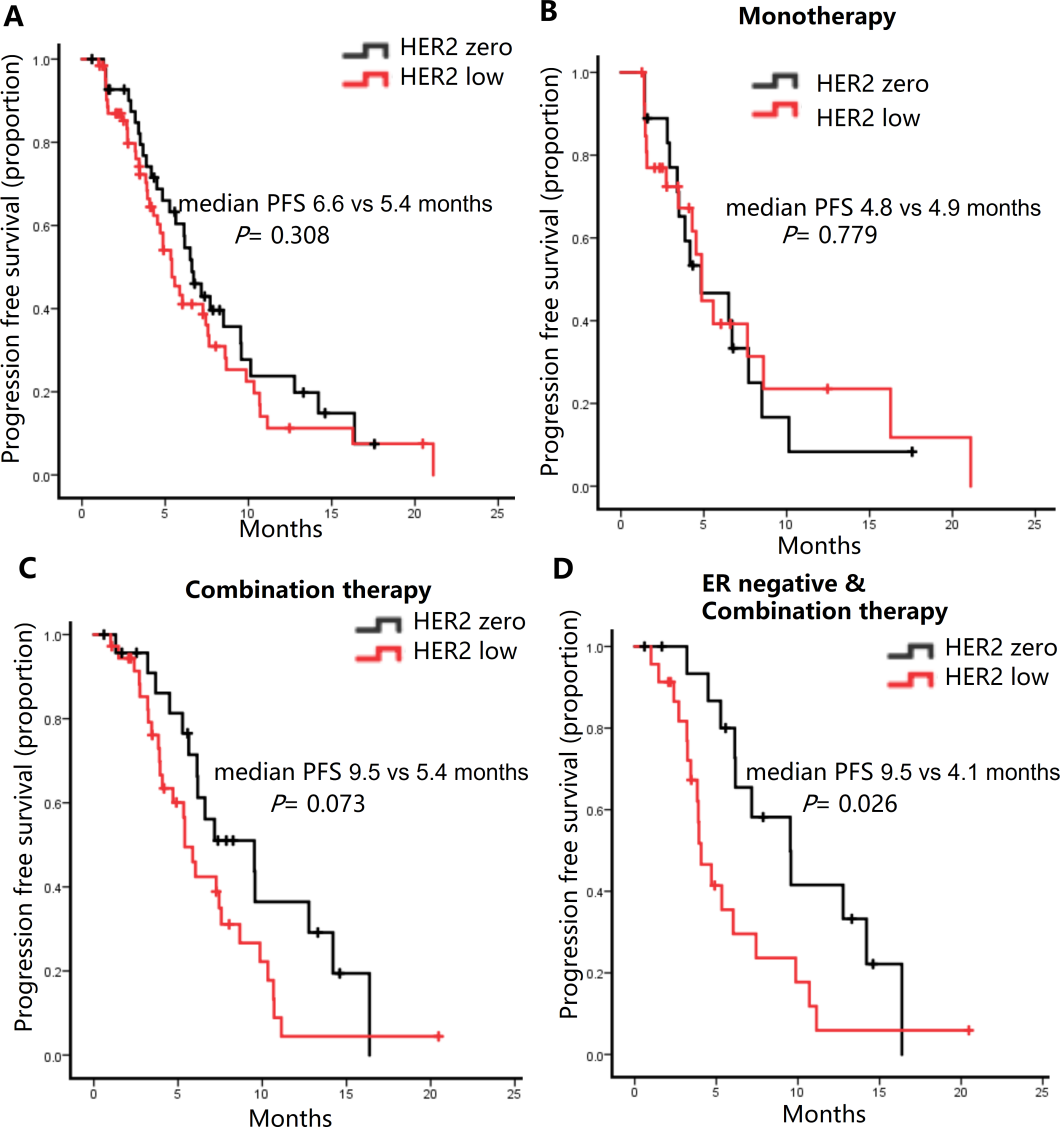
Kaplan-Meier curves of progression-free survival (PFS) in HER2-zero versus HER2-low patients. **(A)** All patients. **(B)** Patients received Eribulin alone. **(C)** Patients receiving Eribulin combined with other drugs. **(D)** ER-negative patients receiving Eribulin combined with other drugs.

In our cohort, 60% of patients (n=63) had HER2-low expression. The response rate and DCR were slightly higher in HER2-zero tumors (31.0% and 98.3%, respectively) compared to HER2-low tumors (28.6% and 85.7%, respectively) (**Table 2**). Unfortunately, neither of them was statistically significant (ORR: P = 0.829; DCR: P = 0.193) (**Supplementary Table 3**). Patients had HER2-low expression demonstrated a slightly shorter mPFS compared to HER2-zero patients (**Figure 3A**). This trend was more pronounced in the combination therapy subgroup (**Figure 3C**), but not eribulin monotherapy group (**Figure 3B**). The mPFS of the HER2-zero group was significantly superior to that of the HER2-low expression group (9.5 vs. 4.1 months, P = 0.026) among estrogen receptor (ER)-negative patients receiving combination therapy (**Figure 3D**).

#### Factors associated with PFS

Univariate and multivariate analyses were performed to explore factors associated with PFS (**Table 3**). Eribulin as third-line or later chemotherapy was associated with lower PFS in the univariate analysis (HR 1.630; 95% CI 1.021-2.601; P = 0.041), a finding confirmed in the multivariate analysis (HR 2.379; 95% CI 1.049-5.398; P = 0.038). Multivariable Cox regression analysis indicated that combination therapy with a PD-1 inhibitor or chemotherapy was an independent risk factor for PFS (HR 0.670; 95% CI 0.498-0.901; P = 0.008).

**Table 3:** Log-rank and Cox multivariate analysis of factors associated with progression free survival.

Characteristic	HR (95% CI)	Log-rank analysis P-value	HR (95% CI)	Cox multivariate analysis P-value
Age (years) >60 vs ≤60	0.894 (0.505-1.582)	0.700	0.721 (0.363-1.432)	0.350
ER Status Positive vs negative	0.914 (0.568-1.469)	0.710	0.663 (0.366-1.200)	0.175
HER2 Status Low vs Zero	1.278 (0.796-2.051)	0.310	1.399 (0.841-2.327)	0.196
Ki67 (10%) >20 vs ≤20	1.019 (0.602-1.726)	0.944	1.111 (0.608-2.032)	0.732
Chemotherapy lines containing Eribulin 3rd/later vs 1st/2nd	1.630 (1.021-2.601)	0.041	2.379 (1.049-5.398)	0.038
Previous therapy (Taxanes)	1.258 (0.788-2.008)	0.336	0.675 (0.315-1.447)	0.312
Previous therapy (Capecitabine)	1.314 (0.806-2.142)	0.274	1.175 (0.601-2.297)	0.637
Previous therapy (Vinorelbine)	1.114 (0.646-1.922)	0.698	0.924 (0.470-1.817)	0.819
Previous therapy (Gemcitabine)	1.200 (0.664-2.168)	0.547	0.856 (0.425-1.724)	0.663
Liver metastasis	0.884 (0.552-1.417)	0.610	0.936 (0.519-1.687)	0.826
Lung metastasis	1.210 (0.761-1.925)	0.420	1.389 (0.803-2.400)	0.240
Bone metastasis	0.949 (0.594-1.516)	0.825	0.962 (0.564-1.640)	0.886
Brain metastasis	0.771 (0.403-1.477)	0.433	0.701 (0.334-1.469)	0.346
Pleura metastasis	1.150 (0.629-2.104)	0.650	0.865 (0.442-1.694)	0.672
Combine with PD-1 inhibitor or chemotherapy vs monotherapy or combine with anti-angiogenesis therapy	0.752 (0.586-0.966)	0.026	0.670 (0.498-0.901)	0.008

### Safety

The most common AEs were leukopenia (72.4%), neutropenia (66.7%), anemia (54.3%), and liver function damage (51.4%) (**Table 4**). Common grade 3 or 4 adverse events reported in more than 10% of patients were neutropenia (40.0%) and leukopenia (24.8%). No significant albumin decreases or renal impairments occurred during the study. All adverse events were manageable and could be improved after corresponding treatment without treatment-related deaths.

**Table 4:** Adverse events.

Adverse events	All Grades (%)	Grade 1-2 (%)	Grade 3-4 (%)
Leukopenia	76 (72.4)	50 (47.6)	26 (24.8)
Neutropenia	70 (66.7)	28 (26.7)	42 (40.0)
Anemia	57 (54.3)	52 (49.8)	5 (4.8)
Thrombocytopenia	34 (32.4)	28 (26.7)	6 (5.7)
Hypoproteinemia	21 (20.0)	21 (20.0)	0 (0)
Transaminitis	54 (51.4)	51 (48.5)	3 (2.9)
renal function damage	4 (3.8)	1 (1.2)	0 (0)

## Discussion

Our study provides crucial real-world evidence on the efficacy and safety of eribulin in the treatment of advanced breast cancer. While eribulin has already shown favorable response rates and clinical benefits for metastatic breast cancer patients in retrospective studies (6, 23), our findings further expand on this knowledge. The median PFS of our study cohort was 6.0 months, notably longer than the reported median PFS of 3–4 months in previous phase 3 clinical trials (24–26). This divergence can be attributed to several key factors. Firstly, prior phase 3 trials predominantly enrolled patients who had received at least three lines of treatment, whereas our study included a significant proportion of patients treated in the first or second-line setting. Intriguingly, our study also reveals that the use of eribulin in the first or second-line setting represents an independent prognostic factor, emphasizing the importance of early-stage intervention. Secondly, previous trials typically evaluated eribulin as a monotherapy, while a subset of patients in our study received combination treatments. The synergistic effects of combining eribulin with other agents, such as PD-1 inhibitor or chemotherapy drugs, may enhance its anti-tumor activity, thereby contributing to the extended PFS observed in our real-world cohort. These distinctions underscore the importance of considering treatment lines and regimens when interpreting Eribulin’s efficacy data. They also highlight the potential benefits of incorporating this agent earlier in the treatment sequence and leveraging combination strategies for advanced breast cancer patients, which could pave the way for more effective and personalized therapeutic approaches.

Notably, significant differences in PFS were observed among different molecular subtypes. Specifically, ER-positive or HER2-zero patients treated with eribulin in the first or second line of therapy achieved median PFS of 10.1 and 9.6 months, respectively. This finding aligns with the current biological understanding, as hormonal pathways may interact with eribulin’s mechanism of action, which targets microtubule dynamics (27). Eribulin’s disruption of microtubule function may synergize with endocrine-based therapies in ER-positive tumors, potentially enhancing its therapeutic efficacy. These results suggest that Eribulin could be a viable and effective early-line treatment option for ER-positive/HER2-zero patients, potentially delaying disease progression and improving quality of life. Regrettably, these differences did not reach statistical significance, likely due to the small sample size of the study population.

Our analysis contributes to the evolving understanding of HER2-low tumors within the spectrum of breast cancer by highlighting their distinct clinical outcomes compared to HER2-zero tumors. Our study results revealed that HER2-low metastatic breast cancer patients exhibited a marginally shorter median PFS, especially within the ER-negative subgroup receiving combination therapy. Paradoxically, this observation suggests that HER2-low patients may demonstrate a superior response to Eribulin chemotherapy. In a retrospective study of advanced breast cancer, a total of 79 patients were administered Eribulin. Among them, 35 patients were categorized as HER2-null, and 44 patients had HER2-low tumors. The analysis of survival outcomes indicated that there were no significant differences in OS or PFS between the HER2-null and HER2-low groups that received Eribulin treatment. The HER2-null and HER2-low patient groups showed ORRs of 22.5% and 9.1%, respectively (p = 0.09). In the subgroup of hormone receptor-positive patients treated with Eribulin, the disparity was even more evident: the ORR in the HER2-null group reached 32.0%, while in the HER2-low group it was only 10.5%, and this difference was statistically significant (p = 0.03) (28). Our own research further demonstrated that in terms of treatment response, the ORR (31.0% vs 28.6%) and DCR (95.2% vs 85.7%) were higher in HER2-zero patients compared to the HER2-low group. However, these differences did not reach statistical significance. This disparity in ORRs strongly emphasizes the necessity for further exploration of the biological mechanisms that underlie the differential response to Eribulin. The unique molecular profiles of HER2-zero and HER2-low tumors are likely to account for their varying sensitivities to microtubule-targeting agents such as Eribulin.

Compared with HER2-zero tumors, the HER2-low subtype exhibits distinct pathological and molecular features, manifesting as fewer grade III tumors, lower Ki-67 expression levels, and reduced incidence of TP53 mutations (29, 30). Moreover, the HER2-low subtype is associated with a lower rate of lymph node metastasis (31). Breast cancer patients have been demonstrated that among HER2-low patients, there is a higher proportion of hormone receptor positive cases (30, 32). The lower rate of lymph node metastasis and tumor grade may be related to the higher proportion of hormone receptor-positive cases (32).

Additionally, the HER2-low subtype frequently shows persistent activation of the EGFR pathway, encompassing the PI3K/AKT (30, 31, 33, 34) and RAS-RAF-MEK-ERK signaling cascades (35). This activation has the potential to drive tumor cell proliferation, enhance cell survival, and confer resistance to therapeutic interventions, thereby contributing to the unique biological behavior and clinical features of HER2-low tumors. A Chinese multi-omics investigation revealed distinct genomic and proteomic landscapes between HR-positive and HR-negative subgroups within HER2-low tumors. The HR-negative subgroup could be further stratified into two subsets. Basal - like tumors exhibited similarities to HER2-zero disease, whereas non-basal-like HER2-low tumors resembled HER2-positive counterparts. The non-basal-like HER2 - low tumors predominantly comprised the HER2-enriched and luminal androgen receptor subtypes. These tumors were marked by PIK3CA mutations, elevated expression of FGFR4, PTK6, and ERBB4, and activation of lipid metabolic pathways (30).

HER2 expression is not merely a marker for classification and prognosis but also a crucial therapeutic target. Anti-HER2 therapy can significantly improve the survival of patients with HER2-positive breast cancer. New anti-HER2 drugs, such as Trastuzumab deruxtecan, can also notably enhance the prognosis of HER2-low patients, thus making the HER2-low patient group a new classification relevant to treatment (36). The HER2-low status has been significantly correlated with a lower density of tumor-infiltrating lymphocytes (TILs). This reduced infiltration of lymphocytes into the tumor microenvironment indicates that HER2-low tumors may create an immunosuppressive niche, impeding the body’s natural anti-tumor immune mechanisms (37).

In the realm of breast cancer neoadjuvant therapy, a notable paradox has emerged regarding HER2-zero and HER2-low tumors. Among hormone receptor-positive and triple-negative breast cancer patients, those with HER2-zero status exhibit a relatively high pCR rate following neoadjuvant treatment relative to HER2-low. This initial therapeutic success, reflected by the eradication of visible cancer cells at the pathological level, suggests effective tumor shrinkage and treatment sensitivity. However, despite this seemingly favorable short-term outcome, clinical follow-up data reveal a disconcerting reality: the elevated pCR rate in HER2-zero patients fails to translate into tangible improvements in DFS and OS (20, 29, 38–40). This divergence between early treatment response and long-term prognosis challenges conventional assumptions about the predictive value of pCR in guiding patient outcomes, highlighting the complexity of tumor biology beyond initial treatment responsiveness.

These findings underscore the pressing need for a comprehensive reevaluation of existing treatment paradigms for breast cancer. The prognostic implications of specific tumor subtypes, such as HER2-low, remain a subject of considerable uncertainty. Conflicting reports have emerged regarding the clinical outcomes associated with HER2-low tumors. Some investigations indicate potential differences in survival rates, treatment responses, and disease recurrence patterns between HER2-low and HER2-zero breast cancer, hinting at distinct biological behaviors (17, 41, 42). Conversely, other studies fail to demonstrate significant prognostic disparities (34), challenging the notion of HER2-low as a clinically relevant subtype.

HER2 demonstrates significant biological complexity, manifesting not only as intratumoral heterogeneity but also as notable discrepancies in expression levels between primary tumors and metastatic lesions within the same patient (43). During the course of treatment, HER2 expression status exhibits dynamic shifts. The transformation can occur in either direction: from HER2-zero to HER2-low or vice versa, from HER2-low to HER2-zero (40, 44–46). This bidirectional change highlights the remarkable plasticity of HER2 expression, which poses significant challenges for accurate diagnosis, prognosis prediction, and targeted therapeutic strategies.

The limited median PFS achieved with Eribulin monotherapy in advanced breast cancer underscores the urgent need for the development of innovative combination therapies. Our research findings present a somewhat complex picture. On one hand, combination therapy did not lead to a significant enhancement in treatment efficacy, and this could potentially be ascribed to the diversity and heterogeneity of the regimens that were put to the test. Specifically, when Eribulin was combined with anti-angiogenic agents, it resulted in a relatively brief PFS of merely 5.4 months. On the other hand, our study also yields preliminary evidence indicating that pairing Eribulin with either PD-1 inhibitors or other chemotherapeutic agents might possess the potential to extend the PFS in comparison to combinations involving anti-angiogenic agents. This finding aligns with results from phase 3 clinical trials, which have demonstrated improved survival outcomes when checkpoint blockade is added to chemotherapy regimens in patients with advanced PD-L1-positive triple-negative breast cancer (47). Real-world data further support the superiority of Eribulin combination therapies over monotherapy, with one study reporting a statistically significant increase in median PFS from 3.4 to 4.5 months (p = 0.007) (48). Additionally, a multicenter phase II trial showed promising results, with an ORR of 37.0% and a DCR of 87.0% in heavily pretreated advanced Triple-negative breast cancer patients treated with the combination of camrelizumab, apatinib, and Eribulin (49). However, the efficacy of Eribulin-based combination therapies remains inconsistent across studies. For instance, a randomized clinical trial in hormone receptor-positive, HER2-negative metastatic breast cancer patients did not observe significant differences in median PFS or ORR when Eribulin was combined with pembrolizumab compared to Eribulin monotherapy (50).

Collectively, our data suggest that combinations of Eribulin with PD-1 inhibitors or alternative chemotherapy regimens may offer greater promise for prolonging PFS compared to anti-angiogenic combinations. However, further research is needed to identify optimal combination strategies, taking into account patient-specific factors such as tumor subtype, biomarker status, and prior treatment history. Standardized clinical trials with well-defined patient populations are essential to clarify the role of Eribulin-based combination therapies and improve outcomes for patients with advanced breast cancer.

Several limitations should be acknowledged when interpreting the findings of this study. Firstly, the retrospective nature of the study design inherently introduces selection bias, Secondly, the patient population exhibited substantial heterogeneity. Variations in hormone receptor status, treatment modalities, and treatment regimens were observed. These diverse factors could confound the results and make it challenging to draw definitive conclusions about the efficacy of Eribulin in specific patient subgroups. Despite the inclusion of patients from two centers, the overall sample size remained relatively small, particularly when stratified by molecular subtypes. This limited sample size decreased the statistical power of the study, increasing the likelihood of type II errors and potentially masking true associations. Owing to the limited follow-up duration, the OS data failed to reach maturity, precluding us from conducting an analysis of OS outcomes. Given these limitations, the results of this study should be interpreted with caution. While our findings provide valuable insights and generate hypotheses for future research, they need to be validated by large-scale, prospective, randomized clinical trials with well-defined inclusion criteria and homogeneous patient populations.

## Conclusion

The present study indicates that Eribulin presents promising therapeutic effects in advanced breast cancer, particularly when used as first- or second-line monotherapy. Patients with HER2-low expression, especially within the combination therapy subgroup, tended to have a shorter median PFS. Combination therapies incorporating PD-1 inhibitors or chemotherapy showed superior outcomes compared to those with anti-angiogenic agents. The manageable toxicity profile of Eribulin further supports its potential role in precision treatment for HER2-negative advanced breast cancer. These findings warrant additional investigation in well-designed, large-sample, multicenter studies to fully elucidate the clinical implications and optimize treatment strategies.

## Data Availability

The raw data supporting the conclusions of this article will be made available by the authors, without undue reservation.
