# Supplementary material for: Efficacy and safety of Eribulin-based chemotherapy in HER2 negative advanced breast cancer patients: a real-world study
Source: Front Oncol. 2025 Jun 6;15:1499701. doi: 10.3389/fonc.2025.1499701 (PMC12179173; doi:10.3389/fonc.2025.1499701)
Supplement: Supplementary file 1 [file Table1.doc]

**Table 1 Patient characteristics at baseline.**

| **Characteristics** | **Number of patients(%)**  **(N = 105)** |
| --- | --- |
| Median age (years, range) | 53.2(30.0-86.0) |
| ECOG PERFORMANCE-STATUS SCORE | |
| 0 | 77(73.33%) |
| 1 | 22(20.95%) |
| 2 | 6(5.71%) |
| HORMONE RECEPTOR STATUS | |
| ER positive | 63(60.00%) |
| ER negative | 42(40.00%) |
| HER2 status | |
| HER2 zero | 42(40.00%) |
| HER2 low | 63(60.00%) |
| Ki67 status(%) | |
| >20 | 21(20.00%) |
| ≤20 | 84(80.00%) |
| METASTATIC SITES | |
| Liver | 48(45.7%) |
| Lung | 49(46.7%) |
| Bone | 59(56.2%) |
| Brain | 15(14.3%) |
| Pleura | 17(16.2%) |
| NUMBER OF METASTATIC SITES | |
| 1 | 25(23.81%) |
| 2 | 31(29.52%) |
| ≥3 | 49(46.67%) |
| CHEMOTHERAPY LINES CONTAINING ERIBULIN | |
| 1st | 32(30.5%) |
| 2rd | 22(21.0%) |
| 3rd or later | 38(52.1%) |
| PREVIOUS CHEMOTHERAPY DRUG | |
| Taxanes | 58(55.24%) |
| Capecitabine | 34(32.38%) |
| Vinorelbine | 23(21.90%) |
| Gemcitabine | 20(19.05%) |
| REGIMEN | |
| Monotherapy | 45(42.86%) |
| Combine with anti-angiogenesis therapy | 22(20.95%) |
| Combine with PD-1 inhibitor | 38(36.19%) |
| Combine with chemotherapy |  |
